# A novel deep learning algorithm for real-time prediction of clinical deterioration in the emergency department for a multimodal clinical decision support system

**DOI:** 10.1038/s41598-024-80268-7

**Published:** 2024-12-03

**Authors:** Arom Choi, Kwanhyung Lee, Heejung Hyun, Kwang Joon Kim, Byungeun Ahn, Kyung Hyun Lee, Sangchul Hahn, So Yeon Choi, Ji Hoon Kim

**Affiliations:** 1 Department of Emergency medicine, Yonsei University College of Medicine, 50-1 Yonsei-ro, Seodaemun-gu, Seoul, Republic of Korea; 2 Institute for Innovation in Digital Healthcare, Yonsei University, Seodaemun-gu, 50 Yonsei-ro, Seoul, Republic of Korea; 3 AITRICS Corp., 218, Teheran-ro, Gangnam-gu, Seoul, Republic of Korea; 4 Division of Geriatrics, Department of Internal Medicine, Yonsei University College of Medicine, Seoul, Republic of Korea

**Keywords:** Clinical decision support system, Emergency department, Deep learning, Multimodal data Integration, Real-time prediction, Patient deterioration, Prognosis, Medical research, Information technology, Signs and symptoms

## Abstract

The array of complex and evolving patient data has limited clinical decision making in the emergency department (ED). This study introduces an advanced deep learning algorithm designed to enhance real-time prediction accuracy for integration into a novel Clinical Decision Support System (CDSS). A retrospective study was conducted using data from a level 1 tertiary hospital. The algorithm’s predictive performance was evaluated based on in-hospital cardiac arrest, inotropic circulatory support, advanced airway, and intensive care unit admission. We developed an artificial intelligence (AI) algorithm for CDSS that integrates multiple data modalities, including vitals, laboratory, and imaging results from electronic health records. The AI model was trained and tested on a dataset of 237,059 ED visits. The algorithm’s predictions, based solely on triage information, significantly outperformed traditional logistic regression models, with notable improvements in the area under the precision-recall curve (AUPRC). Additionally, predictive accuracy improved with the inclusion of continuous data input at shorter intervals. This study suggests the feasibility of using AI algorithms in diverse clinical scenarios, particularly for earlier detection of clinical deterioration. Future work should focus on expanding the dataset and enhancing real-time data integration across multiple centers to further optimize its application within the novel CDSS.

**Supplementary Information:**

The online version contains supplementary material available at 10.1038/s41598-024-80268-7.

## Introduction

With advances in medical technology, the proliferation of diverse disease observation methods has led to increased complexity of available medical information, and more complex clinical decision-making processes, with diverse outcomes and goals^1,2^. Physicians, especially those in the emergency department (ED), are often tasked with assessing extensive clinical data to formulate comprehensive decisions in a timely manner because of restricted resources in the ED^3^. Among these data sources, electronic health records (EHRs) are vast repositories containing a wide array of clinical information. Artificial intelligence (AI) has emerged as a powerful tool for analyzing EHRs and aiding physicians in clinical decision making. Clinical decision support systems (CDSS) are information systems designed to assist decision-makers and interactively support all phases of an end user’s decision-making process^4,5^. In the clinical setting, the AI-CDSS provides physicians with knowledge, patient-specific information, and recommendations. A CDSS that incorporates AI consists of three key components: (i) the artificial intelligence or machine learning algorithm; (ii) the supporting software platform; and (iii) the hardware infrastructure. The AI algorithm in a CDSS must be robust, ensuring seamless integration into CDSS frameworks by supporting data integration, user-friendly interfaces, and effective decision-making guidance. It should extract clinical information from both free text and images with high precision and recall, thereby supporting complex decision-making processes^4,6^. The algorithm must demonstrate strong performance across diverse patient groups, address uncertainty with probabilistic outputs, and maintain transparency through explainability mechanisms. Evaluating data capture capabilities, learning processes, and initial diagnostic accuracy are critical steps in the development and refinement of AI-driven CDSS. The aim of such systems is not to replace decision-makers, clinicians, patients, and health organizations, but to provide relevant knowledge and support in their decision-making, imitating the decision-making framework of physicians^3^.

During clinical decision-making in the ED, physicians commonly employ hypothetical coding reasoning to reach a medical conclusion^7^. This involves starting with the chief complaint of the patient and asking targeted and relevant questions to gather information. Based on patient responses, physicians construct an initial dataset of key features. Subsequently, a differential diagnosis is formed, and additional clinical features required to confirm or rule out potential diagnoses are determined. Then, the most pertinent features are systematically identified. This process continues iteratively and involves gathering information, reasoning, making decisions, recognizing changes, and revising decisions as necessary. Once the probability of a particular decision reaches a predetermined acceptable level, the process concludes, and the decision is finalized. Interpreting clinical data to identify deteriorating patients in a timely manner is vital in the ED setting as it impacts the quality of care^8^. However, the implementation of an intelligent CDSS for the ED presents challenges that must be addressed. Physicians often prioritize longitudinal insights by focusing on changes over time, rather than raw measurement values. To address this, the CDSS should provide recommendations with longitudinal insights, incorporate trends of repeated measurements, and model the sequential order and temporal distance between clinical events^9^. For targeted real-time deterioration prediction, when new observation data are generated by an end user or physician, it must be possible to make a new prediction every time new data arrives, rather than artificially constructing input data. Hence, patients who require more frequent observation are those whose conditions are predicted to change more critically; therefore, it is important to construct a system that enables more frequent predictions in these patients. Furthermore, efficient resource allocation requires not only frequent but also detailed and timely predictions to effectively prioritize patients by accurately assessing their urgency levels. Considering that a significant portion of hospital data exists in nonstructured formats, analyzing and providing feedback on complex textual and non-textual data remain significant challenges for AI because of the immense volume of data, high dimensionality, and data sparsity inherent to medical datasets^10^. The AI-CDSS must be capable of extracting clinical information from both free text and images with high precision and recall rates, while also supporting decision-making processes. Assessing data capture, learning capabilities, and initial diagnostic accuracy is crucial for the research and development of AI-CDSS.

In this study, we designed a novel algorithm for with natural language processing systems and deep learning (DL) techniques, which can be used commercially while ensuring contextually realistic model performance by employing up-to-date training and testing. We comprehensively evaluated the model’s performance to implement it as a decision-making support to provide methods and es for various clinical applications.

## Methods

### Study design and setting

We constructed models using data gathered from the ED of a level 1 tertiary teaching hospital in South Korea from March 2018 to February 2022. As per the regulations, level 1 and 2 EDs are required to have 24-hour staffing by board-certified emergency physicians. For the prospective analysis, we used data collected from July 2022 to December 2022. Approximately 21% of these patients were subsequently admitted to the hospital from the ED, with an average of 2.5% requiring admission to intensive care units (ICU) each month. Upon arrival at the ED, vital signs and illness severity were evaluated by qualified triage nurses, and patients were directed to the appropriate treatment areas. This study adhered to the principles outlined in the Declaration of Helsinki and was approved by the Institutional Review Board of Severance Hospital Human Research Protection Center (approval nos. 4-2022-0544 and 4-2022-0123). Due to the retrospective nature of the study, the need of informed consent was waived by the Institutional Review Board of Severance Hospital Human Research Protection Center.

### Participant selection

The inclusion criteria were individuals aged > 18 years who presented to the ED. The exclusion criteria included incomplete basic information, documented do-not-resuscitate (DNR) status upon ED admission, cases of out-of-hospital cardiac arrest, instances of death upon arrival, and visits for non-medical purposes. Patients who did not undergo laboratory testing in the ED because ongoing clinical monitoring was deemed unnecessary and those who opted to be discharged after receiving straightforward and prompt treatment were also excluded from the study.

### Data collection

Data were collected using a clinical research analysis portal system within the Digital Healthcare Department of the hospital. Each patient was assigned a randomized identification number for research purposes to anonymously retrieve clinical data. Information on various variables was collected for all ED visitors, including age, sex, method of visit, reason for visit (traumatic or non-traumatic), medical history (hypertension, diabetes mellitus, tuberculosis, hepatitis, allergies, and surgical history), presenting symptoms, Korean Triage and Acuity Scale (KTAS) scores, vital signs (systolic and diastolic blood pressure, heart rate, body temperature, and respiratory rate), and mental status. Changes in vital signs, consent for “do not attempt resuscitation” orders obtained during the ED stay, laboratory test results, electrocardiograms (EKGs) records, and chest radiography conducted in the ED were extracted from EHRs. Regarding the outcome variables, the data included disposition orders for ICU admission by a physician, interventions such as the administration of inotropes and vasopressors, intubation for airway management or mechanical ventilation, occurrence of in-hospital cardiac arrest (IHCA) requiring cardiopulmonary resuscitation, and the corresponding time of these events.

### Data pre-processing

Input features and outcome variables were merged to form a unified data table for each participant. The study period extended from ED arrival, which served as the starting point, to the occurrence of any of the four outcome variables, each serving as an endpoint. This resulted in multiple data labels with varying timelines reflecting the diverse outcomes for each patient. To address outliers in the vital sign data, the upper and lower limits were set for each variable based on the physiological ranges. Subsequently, the dataset was divided into training and test sets in an 8:2 ratio to ensure an adequate amount of data for both training and testing purposes.

Due to the heterogeneous severity and symptoms among ED patients, the list of data collected for each patient varied significantly. Most patients underwent only a subset of laboratory tests without frequent follow-up vital sign monitoring. Only 66% of the patients underwent radiography; hence, missing data were encountered. Therefore, we adopted the Unified Multi-modal Set Embedding (UMSE) structure from our previous work^11^. In addition to the UMSE, the type, time, and value information of the input data features were unadjusted, as collected in the medical records. This implied that the model can discern the novelty of each data input and is unaffected by missing data imputation problems.

**Fig. 1:**
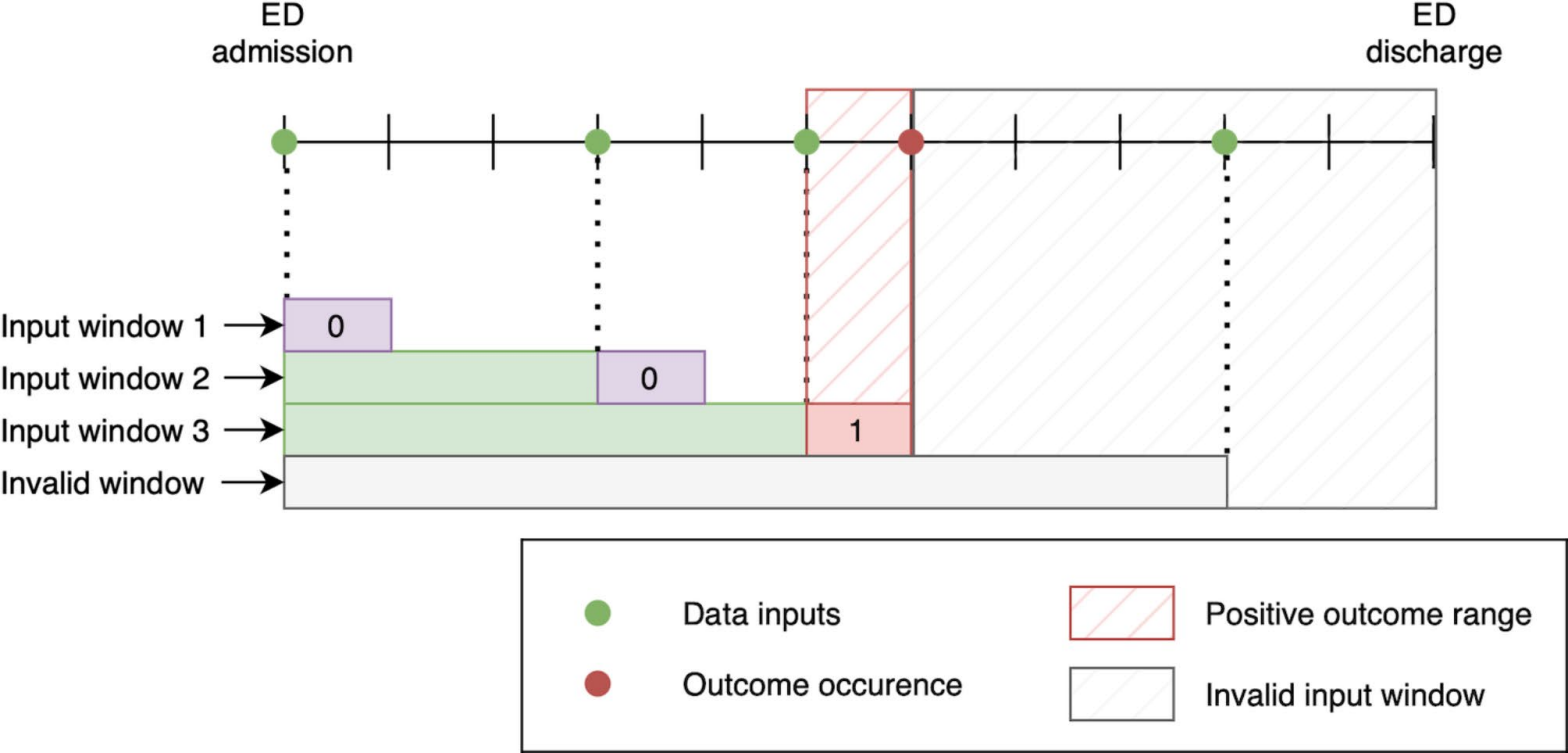
Illustration of the input windows and outcome labeling in a single admission record. Several input windows are extracted based on the time of input acquisition only before the outcome occurrence. First input window contains the input data at the ER admission time and the second one contains inputs from two different acquisition times. In this example, binary outcomes are labeled as positive when outcome occurred within the prediction range of 0 to 1 h. ED, emergency department.

Input data windows were created for each patient, starting from ED admission and extending to subsequent data events. If a patient experienced a critical event, which indicated a positive case, we included all data up to the time of onset; otherwise, we included data up to the time of discharge. For instance, if a medical record is updated three times after admission, it can generate four different input samples/windows: one utilizing only the initial admission information and one for each of the three subsequent events from admission to each event. Binary targets for each input sample were labeled based on whether an outcome occurred within 1–12 h of the reference time, the latest data update, in one hour interval (Fig. 1). As a result, negative input samples can be generated not only from negative outcome cases, but also from positive outcome cases. This led to a significant increase in the total number of input samples, particularly negative samples, compared with the total number of cases. This exacerbated the class imbalance beyond the positive ratio at the case level. To address the issue of class imbalance, two different sampling methods were employed for the retrospective and prospective datasets. Given the large volume of records in the retrospective dataset, we adjusted the class ratio between the negative and positive input windows by oversampling the minority examples. However, the same sampling strategy was not suitable for the prospective dataset owing to its small sample size, because this oversampling method can result in the construction of a mini-batch of input samples from a single record with an extremely long length of stay. Therefore, instead of balancing the classes window-wise, we balanced the classes case-wise in the prospective dataset. This ensured that each mini-batch could be populated with input samples from multiple negative and positive cases.

### Model development

**Fig. 2:**
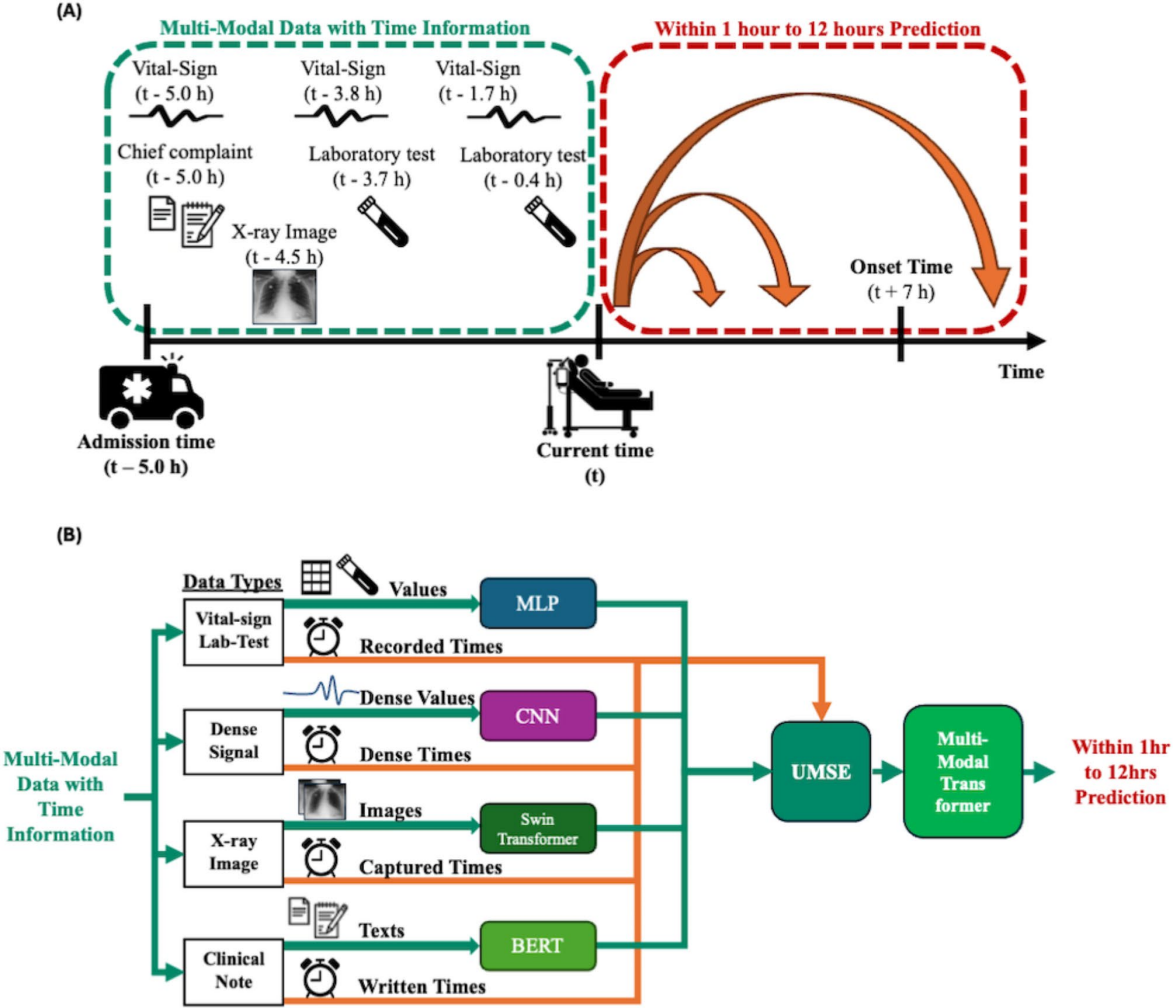
(**A**) Prediction of clinical events with 12 different ranges is achieved by our AI-CDSS, utilizing past multimodal data (**B**) Integration of data encoders and fusion techniques for multimodal predictive modeling using artificial intelligence. MLP, Multilayer Perceptron; CNN, Convolutional Neural Network; BERT, Bidirectional Encoder Representations from Transformers; UMSE, Unified Multi-modal Set Embedding.

In this section, as shown in Fig. 2B, we describe our four modality-specific embedders for sparse time-series tabular data, dense time-series vital signs, radiographs, and textual data. After the modality-specific encoders, the encoder outputs from different modalities are fixed to form 256 hidden dimensions with varying lengths. We then introduce USME from our previous work^11^, which integrates these different modality encoder outputs into structured data while preserving temporal and feature information. Finally, we present the multimodal transformer that handles modality interaction and fusion^12^.

To handle sparse time-series tabular data, such as vital signs and laboratory test results, we employed a multi-layer perceptron (MLP) consisting of two linear layers and an activation function as the modality-specific embedder. Specifically, scalar data, $\:{\text{x}}_{\text{s}\text{p}\text{a}\text{r}\text{s}\text{e}}\:\:\in\:\:{\text{R}}^{1}$, is projected into an embedded vector $\:{\text{e}}_{\text{s}\text{p}\text{a}\text{r}\text{s}\text{e}}\in\:\:{\text{R}}^{256}$ as illustrated in Eq. 1:

1 $$\:{e}_{sparse}=Linea{r}_{256\:}(tanh(Linea{r}_{256}\:\left({x}_{sparse}\:\right)\left)\right)\:$$

For dense time-series data, such as vital signs, we utilized a Convolutional Neural Network (CNN). A CNN was chosen due to its ability to capture local temporal patterns in time-series data, such as changes in vital signs over short time intervals. The dense time-series data, $\:{\text{x}}_{\text{d}\text{e}\text{n}\text{s}\text{e}}\:\in\:\:{\text{R}}^{{\text{t}}_{\text{d}\text{e}\text{n}\text{s}\text{e}}\times\:5}$, comprising 5 vital signs such as respiratory rate, systolic blood pressure, heart rate, diastolic blood pressure, arterial oxygen saturation, where $\:{\text{t}}_{\text{d}\text{e}\text{n}\text{s}\text{e}}$ represents the number of dense data sampling times. This data is transformed into an embedded vector $\:{\text{e}}_{\text{d}\text{e}\text{n}\text{s}\text{e}}\:\in\:\:{\text{R}}^{{\text{t}{\prime\:}}_{\text{d}\text{e}\text{n}\text{s}\text{e}}\times\:256}\:$ where $\:{\text{t}{\prime\:}}_{\text{d}\text{e}\text{n}\text{s}\text{e}}\:\le\:\:{\text{t}}_{\text{d}\text{e}\text{n}\text{s}\text{e}}\:$.

2 $$\:{e}_{dense}=ReLU\left(Conv\right(ReLU\left(Conv\right({x}_{dense}\:\left)\right)\left)\right)\:$$

For processing radiographs, we employed a pre-trained SwinTransformer model^13^ trained on the CheXpert dataset^14^. The SwinTransformer was chosen due to its hierarchical attention mechanism, which enables it to efficiently process images while capturing both global and local patterns in radiographs. The latest chest radiograph, $\:{\text{x}}_{\text{i}\text{m}\text{a}\text{g}\text{e}}\:\in\:\:{\text{R}}^{256\times\:256}$, is selected for each prediction and projected into an embedded vector $\:{\text{e}}_{\text{i}\text{m}\text{a}\text{g}\text{e}}\:\in\:\:{\text{R}}^{256}$ as shown in Eq. 3:

3 $$\:{e}_{image}=Linea{r}_{256}\left(SwinTransformer\right({x}_{image}\:\left)\right)$$

A linear layer is added to project the SwinTransformer output from dimension 768 to 256to standardize the embedded vector size across different modalities.

Lastly, we utilized Bidirectional Encoder Representations from Transformers (BERT) to analyze the textual data, $\:{\text{x}}_{\text{t}\text{e}\text{x}\text{t}}\:\in\:\:{\text{R}}^{{\text{t}}_{\text{t}\text{e}\text{x}\text{t}}}$ (patient chief complaints^15^, patient information, and electrocardiogram reports) where $\:{\text{t}}_{\text{t}\text{e}\text{x}\text{t}}$ indicates the number of tokens in the text data.BERT was selected for its ability to capture the bidirectional context of tokens, which is essential for understanding complex medical narratives. We specifically use the [CLS] token output from BERT, which is designed to capture a summary of the entire input sequence. This [CLS] token is then projected into $\:{\text{e}}_{\text{t}\text{e}\text{x}\text{t}}\:\in\:\:{\text{R}}^{256}$ through a linear layer, as shown in Eq. 4:

4 $$\:{e}_{text}=Linea{r}_{256}\left(BERT\right({x}_{text})\:_{:,Index(CLS)})$$

To integrate these four differently encoded modalities, we adopted the UMSE^11^. UMSE encodes multimodal data by adding time and feature/modality type information to the previously calculated encoder outputs from different modalities, as shown in Eqs. 5 and 6:

5 $$\:{e}_{time}=Linea{r}_{256\:}(tanh(Linea{r}_{256}\:\left({x}_{time}\:\right)\left)\right)\:$$

6 $$\:{e}^{i}={{e}^{i}}_{time}+\:{{e}^{i}}_{value}+{{e}^{i}}_{Feature/Modality}\:\:$$

where $\:{\text{x}}_{\text{t}\text{i}\text{m}\text{e}}$ is the time difference between the data acquisition time and current prediction time.$\:{\text{e}}_{\text{v}\text{a}\text{l}\text{u}\text{e}}$ can be any of $\:{\text{e}}_{\text{s}\text{p}\text{a}\text{r}\text{s}\text{e}},\:{\text{e}}_{\text{d}\text{e}\text{n}\text{s}\text{e}},\:{\text{e}}_{\text{i}\text{m}\text{a}\text{g}\text{e}},\:{\text{e}}_{\text{t}\text{e}\text{x}\text{t}}$, and $\:{\text{e}}_{\text{F}\text{e}\text{a}\text{t}\text{u}\text{r}\text{e}/\text{M}\text{o}\text{d}\text{a}\text{l}\text{i}\text{t}\text{y}}$ is simply obtained from a lookup table similar to word embeddings. The index $\:\text{i}$ refers to the index of acquired data samples.

The UMSE simplified the fusion preparation by eliminating the need for predefined time-interval aggregation or subsequent missing imputation tasks by simply concatenating the embedded vectors, $\:\text{e}$, from different modalities while preserving the time information and feature/modality type of each data.

Finally, the multimodal data unified by the UMSE underwent fusion processing within the multimodal transformer, meticulously capturing both intra- and inter-modality interactions. Here, UMSE output, $$\:\text{e}$$$$\:\in\:\:$$$$\:{\text{R}}^{\text{N}\times\:256}$$where $$\:\text{N}$$ indicates the sum of lengths from all modalities, is fed into the Transformer Encoder. Since $$\:\text{e}$$ already contains temporal information with Eq. 5, no positional encoding step was processed. The transformer then processes these unified embeddings and generates a fused representation.

Multi modal transformer is an actively researched deep learning model, particularly notable for its self-attention module, which calculates the relevance of various elements within the input data^12^. In the medical field, where medical data differ distinctly in the information obtained from each input modality and exhibit interrelationships between modalities, effectively performing fusion tasks is crucial for medical multimodal learning. For example, patient information from vital signs may differ from those obtained from electrocardiograms and chest radiographs; however, physicians integrate all this information for decision-making, thus necessitating an appropriate multi modal fusion model like multimodal transformer.

Our AI-CDSS model was designed to incorporate various multimodal data and precisely evaluate the timing information associated with each data sample to accurately predict patient deterioration over a detailed time range (Fig. 2). The fused representation output from the multimodal transformer is fed into a classifier consisting of two linear layers. Between these layers, we applied batch normalization to stabilize training and a ReLU activation function to introduce non-linearity. The classifier’s output consists of 12 probabilities, each followed by a sigmoid activation function. These probabilities predict whether patient deterioration will occur within 1–12 h from the reference time, at 1-hour intervals (Fig. 2A). We used binary cross-entropy loss for the loss function calculation.

Using retrospective data, we established a two-step model, triage (Step 1), and post-triage (Step 2). In Step 1, we developed a prediction model from the triage stage information, whereas in Step 2, we created a prediction model from the monitoring data after triage.

For the implementation of the LR model, we followed a systematic approach, particularly differentiating between Step 1 and Step 2 models. In Step 1, most of the input features used for training the transformer model were also applied in the LR models. However, DBP was excluded due to its correlation with SBP. Additionally, free-text entries like present illness were not included, as they cannot be processed within the logistic regression framework. For each time window (e.g., 0–1 h, 0–2 h, up to 0–12 h), a separate LR model was developed, resulting in 12 binary prediction models. For Step 2, we expanded the input features to include follow-up data, such as mental status and lab results, in addition to the initial vitals and mental status used in the Step 1 models. To account for the time variance between initial and follow-up data, we calculated the differences between the initial and follow-up vitals and included them as additional input features. In order to handle missing data on follow-up vitals, mental status and lab results, firstly we adopted carry-forward method to fill up missing data with the latest observation, and for those features that have no valid observation we filled up with the mean values of train dataset. We used the scikit-learn package to train the LR models. Most of the hyperparameters were set to the default values, with the exception of increasing the maximum number of iterations to 1000 to ensure model convergence.

For Step 1, we compared the prediction performances of the logistic regression and the bimodal transformer. In Step 2, we compared the performances of the unimodal and trimodal transformer models to further validate the performance of the multimodal model. The unimodal data only included sparse time-series tabular data and the trimodal data then included textual and radiograph data. The target tasks were to predict all four outcomes.

In prospective experiments, we aimed to compare the effectiveness of the models trained with dense time-series tabular data. Specifically, we compared the models trained on sparse time-series tabular data and text data with those trained on sparse time-series tabular data, text data, and dense time-series data. In addition, we discretized the dense time-series data at 10-minute intervals. The target task was to predict circulatory shock.

### Model interpretation

Feature importance analysis was conducted through post hoc feature ablation analysis, utilizing the differences in the area under the receiver operating characteristics curve (AUROC) and area under the precision-recall curve (AUPRC). The UMSE structure enables straightforward creation of modified input samples, systematically eliminating features, including data modality. Additionally, it is important to highlight that, as reported in Liu Z. et al.^13^, models utilizing the UMSE embedding method demonstrate a significant improvement in performance compared to other deep learning models such as Medfuse, HAIM, MT + carry-forward, and MNRIFN^11,16–19^. Considering the variation in data frequency among the features, score comparisons were conducted using a subset of the entire test dataset, including all cases in which the feature of interest was collected at least once. Furthermore, an analysis was conducted on the predictive power of input sequence lengths, assuming that more data inputs were used to obtain more accurate model output returns.

### Outcome measures

Outcome measures included IHCA, circulatory shock, advanced airway, and ICU admission. These four indicators for predicting clinical deterioration in the ED were chosen based on previous studies that identified them as the most critical outcomes^20–23^. IHCA refers to the occurrence of a witnessed or unwitnessed cardiac arrest in the ED for any reason. Circulatory shock refers to the administration of inotropes and vasopressors such as norepinephrine, epinephrine, dopamine, dobutamine, or vasopressin to address shock after adequate fluid administration. Advanced airway was defined as maintenance of the airway with positive pressure ventilation or transitioning from a home ventilator to a conventional ventilator. ICU admission was defined as the decision of the physician to admit a patient to the ICU, irrespective of the diagnosis.

### Analysis

Continuous data are presented as mean values and standard deviations, while categorical data are presented as frequencies and percentages. All statistical tests were two-sided, with the significance level set at *P* < 0.05. Model performance was evaluated using specificity, sensitivity, precision, F1 scores for assessing class imbalance, AUROC, and AUPRC. The evaluation scores were calculated for each prediction window along with the macro-averaged AUROC and AUPRC for all 12 prediction windows. We used the sum of the macro-averaged AUROC and AUPRC scores during the validation step. Following the training step, the model with the highest AUPRC was selected as the optimal model. This decision was made because owing to data imbalance, precision over positive samples is crucial for the real-world application of the model.

## Results

### Study cases and dataset

**Fig. 3:**
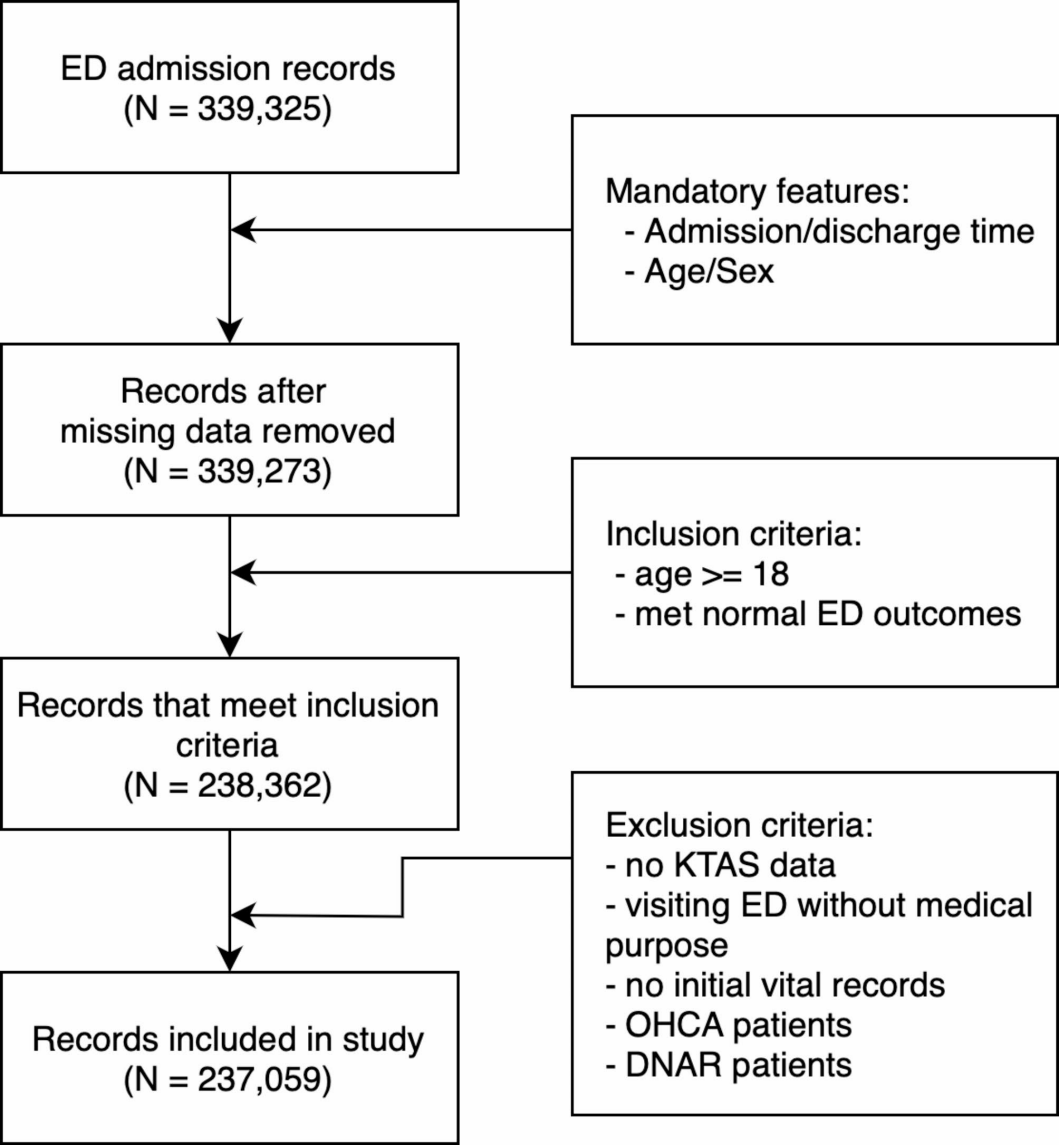
Patient enrollment. ED, Emergency department; KTAS, Korea Triage and Acuity Scale; OHCA, Out-of-Hospital Cardiac Arrest; DNAR, Do Not Attempt Resuscitation.

During the study period, there were 339,324 visits to our emergency department. During the study period, 339,324 visits to our emergency department were recorded. Of these, 35 cases with missing admission and discharge times, and 17 cases with missing sex and age information, were excluded. From these, 248,795 cases involving adults ≥ 18 years with specified ED treatment outcomes were identified. After further excluding visits unrelated to treatment, cases with missing initial vital signs, patients with out-of-hospital cardiac arrest, and patients with DNR orders, the dataset used for the final model development consisted of 237,059 visits (Fig. 3). The dataset was divided into training and test sets at a ratio of 4:1. The baseline clinical characteristics of each set are presented in Table 1.

**Table 1:** Baseline clinical characteristics between training and test set.

Variable	Total	Train	Test	*P*-Value
	(*N* = 237059)	(*N* = 189680)	(*N* = 47379)	
Age (years)	52.9 (19.9)	52.9 (20.0)	52.8 (19.9)	0.223
Sex: Male (n, %)	110,964 (46.8)	88,788 (46.8)	22,176 (46.8)	0.992
Length of stay (n, %)				
0 ~ 12 h	201,263 (84.9)	160,985 (84.9)	40,278 (85.0)	0.327
12 ~ 24 h	23,813 (10.0)	19,135 (10.1)	4678 (9.9)	
24 ~ hrs	11,983 (5.1)	9560 (5.0)	2423 (5.1)	
KTAS (n, %)				
1	2795 (1.2)	2267 (1.2)	528 (1.1)	0.262
2	21,201 (8.9)	16,971 (8.9)	4230 (8.9)	
3	66,664 (28.1)	53,178 (28.0)	13,486 (28.5)	
4	116,396 (49.1)	93,228 (49.2)	23,168 (48.9)	
5	30,003 (12.7)	24,036 (12.7)	5967 (12.6)	
Initial vital signs				
Systolic blood pressure (mmHg, IQR)	134.0 (26.8)	134.0 (26.8)	133.9 (26.9)	0.414
Diastolic blood pressure (mmHg, IQR)	79.7 (15.1)	79.7 (15.1)	79.7 (15.0)	0.634
Heart rate (min^− 1^)	88.6 (19.5)	88.6 (19.5)	88.6 (19.6)	0.403
Respiratory rate (min^− 1^)	17.5 (2.7)	17.5 (2.7)	17.5 (2.7)	0.639
Body temperature (℃)	36.8 (2.1)	36.8 (2.2)	36.8 (2.1)	0.653
Oxygen saturation (%)	97.1 (6.7)	97.1 (6.8)	97.2 (6.4)	0.056
Initial mental status (n, %)				
Alert	226,751 (98.1)	181,449 (98.1)	45,302 (98.2)	0.951
Drowsy	2380 (1.0)	1912 (1.0)	468 (1.0)	
Stupor	1429 (0.6)	1136 (0.6)	293 (0.6)	
Semicoma	290 (0.1)	233 (0.1)	57 (0.1)	
Coma	190 (0.1)	155 (0.1)	35 (0.1)	
Laboratory test (n, %)	170,219 (71.8)	136,179 (71.8)	34,040 (71.8)	
Chest x-ray (n, %)	157,369 (66.4)	125,843 (66.3)	31,526 (66.5)	
Electrocardiogram (n, %)	103,143 (43.5)	82,592 (43.5)	20,551 (43.4)	
Outcomes (n, %)				
In hospital cardiac arrest	414 (0.2)	332 (0.2)	82 (0.2)	0.976
Advanced airway	2129 (0.9)	1706 (0.9)	423 (0.9)	0.913
Circulatory shock	6176 (2.6)	4933 (2.6)	1243 (2.6)	0.793
ICU admission	8375 (3.5)	6709 (3.5)	1666 (3.5)	0.838

KTAS, Korea Triage and Acuity Scale; IQR, Interquartile range; ICU, Intensive Care Unit.

Meanwhile, 230 patients with a total of 245 visits participated in the prospective analysis using continuous data. All cases met the inclusion criteria used in the retrospective study. However, 15 cases without monitoring device data and 36 cases with DNR orders were excluded from the analysis. We used 20% of the total cases as an independent test set, and the baseline clinical characteristics of the training and test sets are presented in Table 2.

**Table 2:** Baseline clinical characteristics between training and test set in the prospective analysis using continuous data.

Variable	Total	Train	Test	*P*-Value
	(*N* = 195)	(*N* = 159)	(*N* = 36)	
Age (years)	70.1 (12.7)	70.7 (12.6)	67.6 (12.8)	0.195
Sex: Male (n, %)	110 (56.4)	90 (56.6)	20 (55.6)	0.992
Length of stay (n, %)				
0 ~ 12 h	73 (37.4)	58 (36.5)	15 (41.7)	0.579
12 ~ 24 h	42 (21.5)	33 (20.8)	9 (25.0)	
24 ~ hrs	80 (41.0)	68 (42.8)	12 (33.3)	
KTAS (n, %)				
1	28 (14.4)	23 (14.5)	5 (13.9)	0.296
2	93 (47.7)	74 (46.5)	19 (52.8)	
3	70 (35.9)	60 (37.7)	10 (27.8)	
4	4 (2.1)	2 (1.3)	2 (5.6)	
5	0 (0.0)	0 (0.0)	0 (0.0)	
Initial vital signs				
Systolic blood pressure (mmHg, IQR)	117.7 (36.7)	118.2 (38.0)	115.9 (30.6)	0.701
Diastolic blood pressure (mmHg, IQR)	69.9 (19.1)	70.0 (19.7)	69.6 (16.6)	0.914
Heart rate (min^− 1^)	102.7 (26.2)	102.1 (27.5)	105.4 (19.8)	0.407
Respiratory rate (min^− 1^)	20.7 (4.9)	21.0 (5.1)	19.7 (4.0)	0.099
Body temperature (℃)	36.7 (1.6)	36.8 (1.0)	36.3 (2.9)	0.326
Oxygen saturation (%)	94.9 (5.5)	94.8 (5.6)	95.4 (5.0)	0.508
Initial mental status (n, %)				
Alert	165 (84.6)	133 (83.6)	32 (88.9)	0.178
Drowsy	18 (9.2)	17 (10.7)	1 (2.8)	
Stupor	8 (4.1)	5 (3.1)	3 (8.3)	
Semicoma	4 (2.1)	4 (2.5)	0 (0.0)	
Coma	0 (0.0)	0 (0.0)	0 (0.0)	
Laboratory test (n, %)	195 (100.0)	159 (100.0)	36 (100.0)	
Electrocardiogram (n, %)	165 (84.6)	133 (83.6)	32 (88.9)	
Outcomes (n, %)				
Circulatory shock	47 (24.1)	39 (24.5)	8 (22.2)	0.939

KTAS, Korea Triage and Acuity Scale; IQR, Interquartile range.

### Model performance

**Fig. 4:**
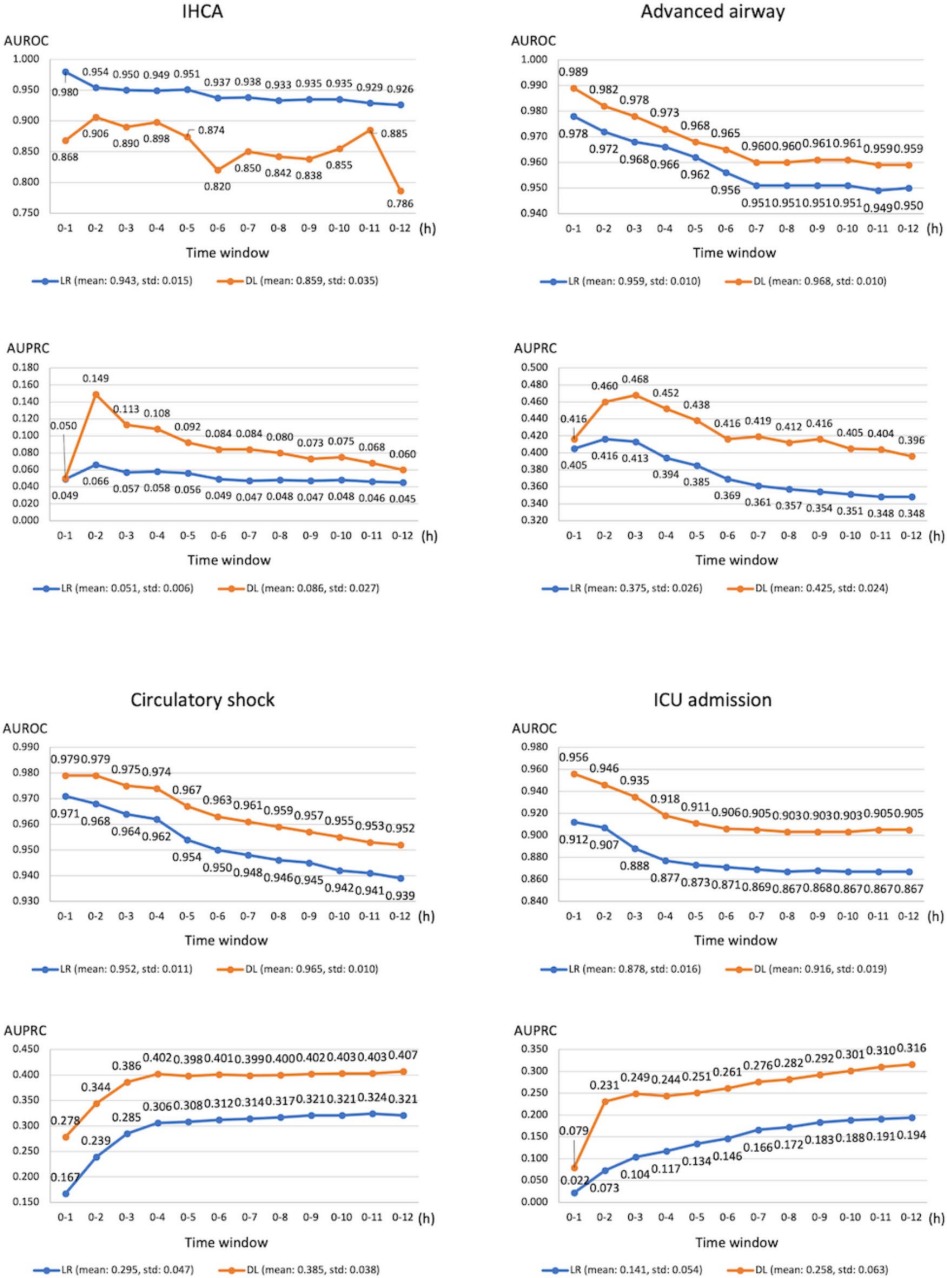
The AUROC and AUPRC scores in each prediction window for the Step 1 (triage) prediction model. AUROC, area under the receiver operating characteristics curve; AUPRC, area under the precision-recall curve; LR, logistic regression; DL, deep learning; IHCA, in hospital cardiac arrest; ICU, intensive care unit.

Figure 4 illustrates the performance of the Step 1 prediction modeling using deep learning for the four critical outcomes across different time windows compared with the conventional logistic regression approach. For the three outcomes other than IHCA, models developed using deep learning had higher AUPRC and AUROC values than those obtained using the logistic regression approach. For IHCA prediction, the AUPRC was higher across all time windows for the methods utilizing deep learning, while the AUROC was lower than the logistic regression approach.

**Fig. 5:**
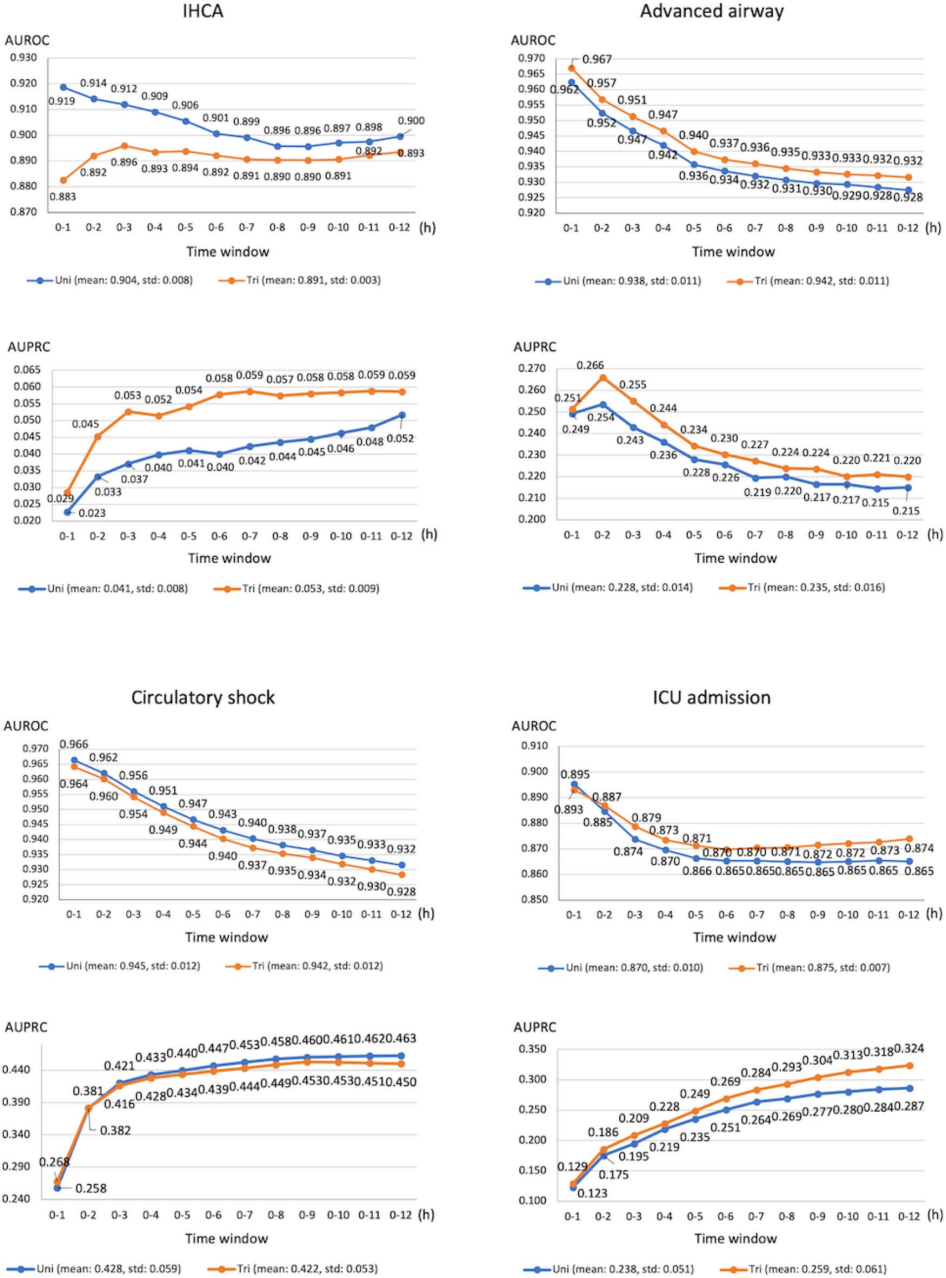
The AUROC and AUPRC scores in each prediction window for the Step 2 (after triage) prediction model. AUROC, area under the receiver operating characteristics curve; AUPRC, area under the precision-recall curve; Uni, unimodal; Tri, trimodal; IHCA, in hospital cardiac arrest; ICU, intensive care unit.

In Fig. 5, the predictive performance of the Step 2 prediction modeling using deep learning for the four critical outcomes is shown across different time windows, comparing the use of a trimodal approach, which includes all forms of clinical information, both structured and unstructured, with a unimodal approach that utilizes only structured data. For IHCA prediction, AUROC values were higher for the unimodal approach than for the trimodal approach but adding modalities increased the AUPRC values. For circulatory shock detection, the performance of the unimodal approach was slightly better than the trimodal approach based on the AUROC and AUPRC. For the remaining outcomes, the trimodal approach demonstrated higher AUPRC and AUROC values. The confidence intervals were provided as supplementary Table 1.

### Clinical validation of the model predictions

**Fig. 6:**
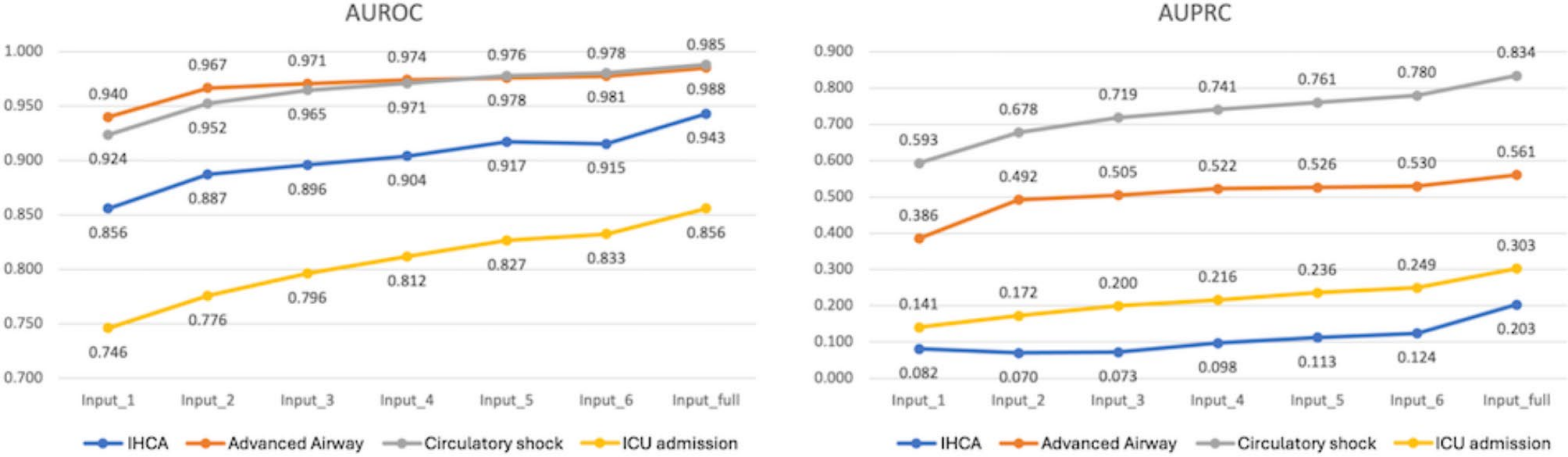
Change of model prediction power alongside the length of the input sequence. AUROC, area under the receiver operating characteristics curve; AUPRC, area under the precision-recall curve; IHCA, in hospital cardiac arrest; ICU, intensive care unit.

**Fig. 7:**
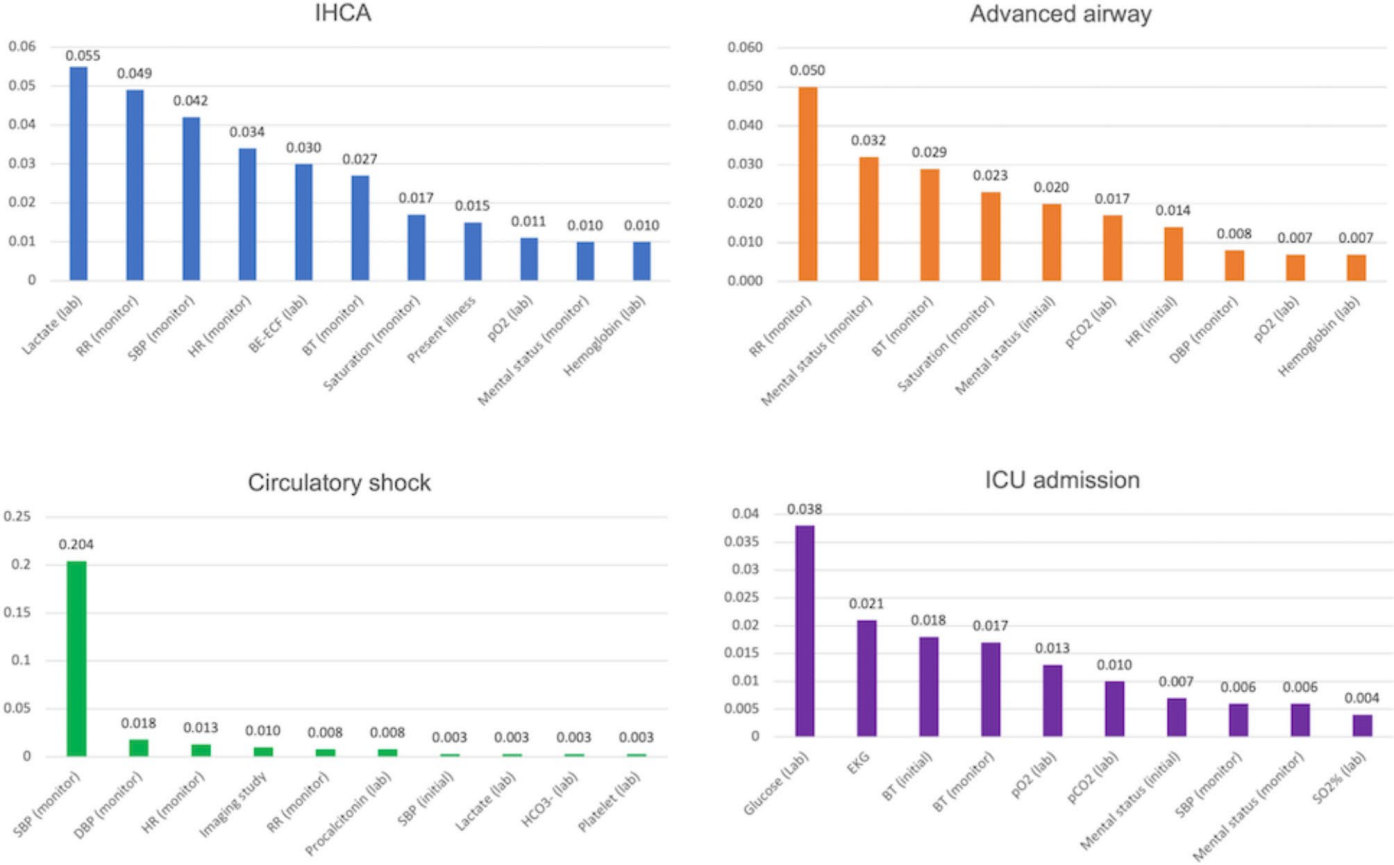
Top 10 important features in the four outcome measures. IHCA, in hospital cardiac arrest; ICU, intensive care unit; RR, respiratory rate; SBP, systolic blood pressure; HR, heart rate; BE-ECF, base excess-extracellular fluid; BT, body temperature; DBP, diastolic blood pressure; EKG, echocardiogram; SO2, arterial oxygen saturation.

The results of additional analyses conducted to evaluate the clinical validity of the present model is shown in Fig. 6. The AUROC and AUPRC increased for all outcomes when the model used sequential clinical information of patients over a longer time period for critical event predictions (Fig. 6). We also evaluated the importance of input variables in predicting each critical outcome using the trimodal model, and presented the items with high importance for each outcome in Fig. 7. In predicting advanced airway application, the patient’s respiratory rate and level of consciousness were identified as the two variables with high feature importance. For circulatory shock prediction, the patient’s systolic and diastolic blood pressure, and heart rate were found to have high feature importance. In addition, blood test values (procalcitonin, lactate, and platelet count) that serve as indicators for predicting sepsis were also included among the top 10 variables with high importance in predicting circulatory shock.

### Prospective analysis using continuous data

**Fig. 8:**
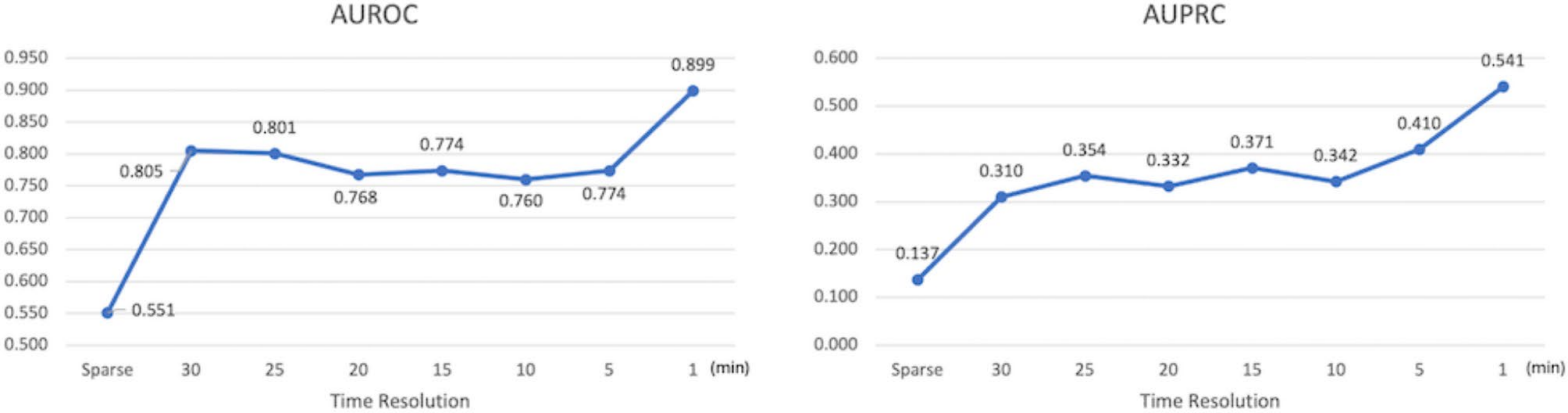
The AUROC and AUPRC scores alongside the time resolution of input data. AUROC, area under the receiver operating characteristics curve; AUPRC, area under the precision-recall curve.

Figure 8 and supplementary Table 2 demonstrate how the predictive performance of our model, which is used to predict circulatory shock, varies with time resolution when applied to a prospectively collected dense dataset. It was observed that the ability of the model to predict the progression to circulatory shock in patients improved as continuous vital sign information in waveform format was input at shorter intervals.

## Discussion

In the present study, the proposed algorithm for an AI-CDSS leveraged optimized deep learning technologies designed to reflect the decision-making process of physicians who must predict critical outcomes with a continuously updated, erratic, and continuous influx of patient information in the ED. Also, it is designed to meet the core characteristics of CDSS itself. It integrates multimodal data (vital signs, lab results, clinical notes) to provide real-time, patient-specific recommendations fitting into clinical workflows by integrating with the emergency physician’s actual process, offering probabilistic outputs to handle uncertainty, and ensuring transparency through explainability mechanisms. Previous studies that presented AI algorithms for predicting critical outcomes using retrospectively collected EHR data have a blind spot in not reflecting the temporal information at the point of data acquisition^17,24–28^. These approaches are insufficient for practical implementation in CDSS because they develop algorithms by fixing the types of required inputs and applying imputation methods such as carry forward or time quantization when missing values occur^3^. The clinical environment of the ED is characterized by frequent occasions where inputs necessary for predicting severe deterioration are not available, and it takes a long time to collect all the information, necessitating the best decision-making with limited information. Moreover, emergency physicians routinely update their clinical decisions based on clinical information accumulated over time following the course of admission of a patient. Therefore, it is crucial that models be designed to reflect the sequential timeline over which various clinical data on patients are obtained. As a solution, we encoded the EHR input data into embedding vectors based on the types of information, such as systolic blood pressure, chest radiograph, present illness, time when the information was acquired, and its value^11^. Through this approach, our model demonstrated the ability to make predictions by fully reflecting the value and changes in the entered information and considering the temporal flow of information acquired after admission, similar to the conventional clinical practice of emergency physicians.

From the perspective of a physician, when predicting patient deterioration, the clinical information of patients must be clearly distinguished by the modality in which they are acquired while also understanding the relationships between each modality. For example, the information obtained by measuring a patient’s vital signs and the information visible through a chest radiograph should be recognized as originating from different modalities; however, physicians integrate these pieces of information to make clinical decisions. We extracted information from each modality to reflect these clinical characteristics then integrated this information considering both inter-modality and modality-outcome correlation, while also handling the problem of missing input data. This approach aimed to not only accommodate the individual characteristics of structured datasets but also those of unstructured datasets in image and textual forms, thereby deriving integrated predictions^11^. The validation results of the predictive model proposed in our study demonstrate the clinical benefits of using a trimodal approach that leverages unstructured data to predict ICU admission and advanced airways. In addition, it was found that not only the initial vital signs of the patients but also the vital signs monitored over time showed high importance in predicting critical outcomes. Furthermore, the study confirmed through prospective validation that values automatically acquired in waveform and represented in continuous form with reduced time resolution significantly contributed to improved prediction accuracy. Since the sample size for the prospective analysis using continuous data is small, it may not have the same level of power as results derived from a retrospective dataset. However, these findings suggest that the use of unstructured data is clinically beneficial.

Given the practical application of the predictive model in real-time clinical decision-making rather than academic research, our focus was on crafting an efficient end-to-end system, ensuring it offers substantial value beyond currently available systems, both in terms of usability and performance, while requiring minimal pre- and post-processing of real-world EHR data. Integrating AI into clinical workflows requires more than just achieving high predictive accuracy. It is essential for the AI-powered CDSS to effectively enhance situation awareness for healthcare professionals. While many studies show that AI-based algorithms can outperform clinicians in specific tasks, real-world application demands that these systems present predictions in a meaningful, actionable manner. For example, models like the KTAS have been extensively validated and are effective in many emergency department scenarios^29,30^. Our deep learning model, when compared to KTAS, showed strong performance across key outcomes, underscoring the value of multimodal approaches that integrate both structured and unstructured data. Furthermore, prior studies have been conducted to predict critical clinical outcomes, such as acute deterioration or in-hospital mortality, and actual cases leading to the development of a CDSS are exceedingly rare. To achieve this, it is essential that the clinical information obtained through patient care is entered by medical staff and integrated with the model in real time, and for various types of data occurring in the clinical setting to be captured automatically without the need for manual input^31^. Considering usability, we did not use radiological interpretations that were retrospectively processed by humans as inputs for our model, nor did we artificially categorize the medical histories or symptoms of patients into discrete variables. Instead, image data were input in their original form, and information regarding medical history and symptoms was utilized in the raw textual state. In addition, similar to the proactive input of vital signs in a waveform format through IoT devices for model utilization, integrating various input modalities capable of real-time data entry can significantly enhance the system. This integration is expected to improve the usability and applicability of the model in dynamic clinical environments.

This study presents several avenues for future research. Despite the comprehensive collection of data from ED patients across various input modalities, this study primarily relied on a retrospective design conducted at a single center. Therefore, a large-scale prospective multicenter study is essential to validate the usability and applicability of the proposed model as implemented software in a real-world clinical setting to ensure patient safety and garner the trust of clinicians. Furthermore, incorporating real-time data such as vital signs from patient monitoring devices into an automated data collection system is crucial for both clinician usability and the predictive power of the models. Therefore, to ensure the effectiveness of the prediction model developed in this study, it is necessary to develop a system that can integrate data from various modalities in real-time. Furthermore, while our study demonstrates the effectiveness of the proposed deep learning model, we did not perform a direct comparison with other deep learning approaches. Future work should explore comparative analyses with alternative deep learning methods to further validate the superiority of our approach. Finally, since the image data in the model was inputted in its original form, there remains a potential concern in real-world applications. The possibility that prior clinical information could influence AI interpretation is a recognized issue. Future research and validation are necessary to develop a dedicated module to mitigate this risk.

In summary, the proposed algorithm for an AI-CDSS can handle sparse input data, integrate patient information from diverse data modalities, and precisely predict outcomes aligned with the schedules of ED physicians. However, further prospective validation studies are necessary to address the remaining challenges, particularly by enhancing the model performance for positive cases, to establish the clinical utility of the AI-CDSS in supporting clinical-decision making for ED physicians.

## Electronic supplementary material

Below is the link to the electronic supplementary material.

Supplementary Material 1

## Data Availability

The datasets analyzed during the current study are available from the corresponding author on reasonable request.
