# Supplementary material for: A novel deep learning algorithm for real-time prediction of clinical deterioration in the emergency department for a multimodal clinical decision support system
Source: Sci Rep. 2024 Dec 3;14:30116. doi: 10.1038/s41598-024-80268-7 (PMC11615388; doi:10.1038/s41598-024-80268-7)
Supplement: Supplementary file 1 — Supplementary Material 1 [file 41598_2024_80268_MOESM1_ESM.docx]

| **Model**  **architecture** | |  |  | **Multi Within Ranges Prediction Performance** | | | | | | | | | | | | | |
| --- | --- | --- | --- | --- | --- | --- | --- | --- | --- | --- | --- | --- | --- | --- | --- | --- | --- |
| **data** | **model** | **Prediction**  **time** |  | **mean** | **std** | **0-1** | **0-2** | **0-3** | **0-4** | **0-5** | **0-6** | **0-7** | **0-8** | **0-9** | **0-10** | **0-11** | **0-12** |
|  |  | Step1  (Triage) | class ratio (%) | | | 0.04 | 0.07 | 0.08 | 0.09 | 0.10 | 0.11 | 0.12 | 0.13 | 0.13 | 0.13 | 0.14 | 0.14 |
| Uni  modal_A | Logistic  regression |  | AUC | 0.943 | 0.015 | 0.980 (0.938 - 1.000) | 0.954 (0.905 - 1.000) | 0.950 (0.902 - 0.998) | 0.949 (0.904 - 0.994) | 0.951 (0.908 - 0.994) | 0.937 (0.892 - 0.982) | 0.938 (0.894 - 0.982) | 0.933 (0.889 - 0.977) | 0.935 (0.892 - 0.978) | 0.935 (0.892 - 0.978) | 0.929 (0.886 - 0.972) | 0.926 (0.882 - 0.970) |
|  |  |  | APR | 0.051 | 0.006 | 0.049 (0.007 - 0.272) | 0.066 (0.018 - 0.215) | 0.057 (0.015 - 0.190) | 0.058 (0.017 - 0.179) | 0.056 (0.017 - 0.171) | 0.049 (0.015 - 0.151) | 0.047 (0.014 - 0.144) | 0.048 (0.015 - 0.141) | 0.047 (0.015 - 0.138) | 0.048 (0.015 - 0.139) | 0.046 (0.015 - 0.133) | 0.045 (0.015 - 0.131) |
| Bi  modal_A | Multimodal  Transformer |  | AUC | 0.859 | 0.035 | 0.868 (0.769 - 0.967) | 0.906 (0.838 - 0.974) | 0.890 (0.823 - 0.957) | 0.898 (0.836 - 0.960) | 0.874 (0.809 - 0.939) | 0.820 (0.751 - 0.889) | 0.850 (0.787 - 0.913) | 0.842 (0.776 - 0.900) | 0.838 (0.795 - 0.915) | 0.855 (0.795 - 0.915) | 0.885 (0.832 - 0.938) | 0.786 (0.720 - 0.852) |
|  |  |  | APR | 0.086 | 0.027 | 0.050 (0.007 - 0.272) | 0.149 (0.064 - 0.310) | 0.113 (0.045 - 0.256) | 0.108 (0.045 - 0.239) | 0.092 (0.036 - 0.214) | 0.084 (0.034 - 0.193) | 0.084 (0.035 - 0.189) | 0.080 (0.029 - 0.170) | 0.073 (0.031 - 0.173) | 0.075 (0.031 - 0.173) | 0.068 (0.027 - 0.161) | 0.060 (0.023 - 0.150) |
|  |  | Step2  (After  -Triage) | class ratio (%) | | | 0.10 | 0.15 | 0.19 | 0.22 | 0.25 | 0.28 | 0.30 | 0.31 | 0.33 | 0.34 | 0.36 | 0.37 |
| Uni  modal  _C | Logistic  regression |  | AUC | 0.889 | 0.005 | 0.884 (0.862 - 0.906) | 0.898 (0.881 - 0.915) | 0.899 (0.884 - 0.914) | 0.890 (0.875 - 0.905) | 0.890 (0.876 - 0.904) | 0.883 (0.870 - 0.896) | 0.885 (0.872 - 0.898) | 0.884 (0.871 - 0.897) | 0.885 (0.873 - 0.897) | 0.888 (0.876 - 0.900) | 0.890 (0.878 - 0.902) | 0.893 (0.882 - 0.904) |
|  |  |  | APR | 0.035 | 0.009 | 0.016 (0.007 - 0.035) | 0.024 (0.014 - 0.040) | 0.027 (0.017 - 0.042) | 0.034 (0.023 - 0.049) | 0.037 (0.027 - 0.051) | 0.036 (0.026 - 0.049) | 0.038 (0.028 - 0.051) | 0.038 (0.028 - 0.051) | 0.039 (0.029 - 0.051) | 0.040 (0.031 - 0.052) | 0.043 (0.033 - 0.055) | 0.046 (0.036 - 0.058) |
| Uni  modal_B | Unimodal  Transformer |  | AUC | 0.904 | 0.008 | 0.919 (0.900 - 0.938) | 0.914 (0.898 - 0.930) | 0.912 (0.897 - 0.927) | 0.909 (0.895 - 0.923) | 0.906 (0.893 - 0.919) | 0.901 (0.888 - 0.914) | 0.899 (0.887 - 0.911) | 0.896 (0.884 - 0.908) | 0.896 (0.884 - 0.908) | 0.897 (0.886 - 0.908) | 0.898 (0.887 - 0.909) | 0.900 (0.889 - 0.911) |
|  |  |  | APR | 0.041 | 0.008 | 0.023 (0.012 - 0.044) | 0.033 (0.021 - 0.051) | 0.037 (0.025 - 0.054) | 0.040 (0.028 - 0.056) | 0.041 (0.030 - 0.056) | 0.040 (0.030 - 0.054) | 0.042 (0.032 - 0.056) | 0.044 (0.034 - 0.057) | 0.045 (0.035 - 0.058) | 0.046 (0.036 - 0.059) | 0.048 (0.038 - 0.061) | 0.052 (0.041 - 0.065) |
|  |  |  | class ratio (%) | | | 0.10 | 0.15 | 0.18 | 0.21 | 0.24 | 0.27 | 0.29 | 0.30 | 0.32 | 0.33 | 0.34 | 0.35 |
| Tri modal | Multimodal  Transformer |  | AUC | 0.891 | 0.003 | 0.883 (0.861 - 0.905) | 0.892 (0.875 - 0.909) | 0.896 (0.881 - 0.911) | 0.893 (0.879 - 0.907) | 0.894 (0.881 - 0.907) | 0.892 (0.879 - 0.905) | 0.891 (0.879 - 0.903) | 0.890 (0.878 - 0.902) | 0.890 (0.878 - 0.902) | 0.891 (0.880 - 0.902) | 0.892 (0.881 - 0.903) | 0.893 (0.882 - 0.904) |
|  |  |  | APR | 0.053 | 0.009 | 0.029 (0.016 - 0.051) | 0.045 (0.031 - 0.065) | 0.053 (0.039 - 0.072) | 0.052 (0.039 - 0.069) | 0.054 (0.041 - 0.070) | 0.058 (0.046 - 0.074) | 0.059 (0.047 - 0.074) | 0.057 (0.045 - 0.071) | 0.058 (0.046 - 0.072) | 0.058 (0.047 - 0.072) | 0.059 (0.048 - 0.073) | 0.059 (0.048 - 0.072) |

**Supplementary table 1. The AUROC and AUPRC scores in each prediction window with 95% confidence interval**

**(A) In hospital cardiac arrest (B) Advanced airway (C) Circulatory shock (D) Intensive care unit admission**

(A)

| **Model**  **architecture** | |  |  | **Multi Within Ranges Prediction Performance** | | | | | | | | | | | | | |
| --- | --- | --- | --- | --- | --- | --- | --- | --- | --- | --- | --- | --- | --- | --- | --- | --- | --- |
| **data** | **model** | **Prediction**  **time** |  | **mean** | **std** | **0-1** | **0-2** | **0-3** | **0-4** | **0-5** | **0-6** | **0-7** | **0-8** | **0-9** | **0-10** | **0-11** | **0-12** |
|  |  | Step1  (Triage) | class ratio (%) | | | 0.44 | 0.53 | 0.58 | 0.63 | 0.66 | 0.69 | 0.71 | 0.72 | 0.73 | 0.74 | 0.76 | 0.76 |
| Uni  modal_A | Logistic  regression |  | AUC | 0.959 | 0.010 | 0.978 (0.964 - 0.992) | 0.972 (0.958 - 0.986) | 0.968 (0.953 - 0.983) | 0.966 (0.952 - 0.980) | 0.962 (0.947 - 0.977) | 0.956 (0.940 - 0.972) | 0.951 (0.935 - 0.967) | 0.951 (0.935 - 0.967) | 0.951 (0.935 - 0.967) | 0.951 (0.935 - 0.967) | 0.949 (0.933 - 0.965) | 0.950 (0.934 - 0.966) |
|  |  |  | APR | 0.375 | 0.026 | 0.405 (0.340 - 0.473) | 0.416 (0.356 - 0.478) | 0.413 (0.356 - 0.472) | 0.394 (0.340 - 0.451) | 0.385 (0.333 - 0.440) | 0.369 (0.318 - 0.423) | 0.361 (0.311 - 0.414) | 0.357 (0.308 - 0.409) | 0.354 (0.305 - 0.406) | 0.351 (0.303 - 0.403) | 0.348 (0.300 - 0.399) | 0.348 (0.301 - 0.399) |
| Bi  modal_A | Multimodal  Transformer |  | AUC | 0.968 | 0.010 | 0.989 (0.979 - 0.999) | 0.982 (0.970 - 0.994) | 0.978 (0.966 - 0.990) | 0.973 (0.960 - 0.986) | 0.968 (0.954 - 0.982) | 0.965 (0.951 - 0.979) | 0.960 (0.945 - 0.975) | 0.960 (0.945 - 0.975) | 0.961 (0.947 - 0.975) | 0.961 (0.947 - 0.975) | 0.959 (0.945 - 0.973) | 0.959 (0.926 - 0.992) |
|  |  |  | APR | 0.425 | 0.024 | 0.416 (0.351 - 0.484) | 0.460 (0.399 - 0.522) | 0.468 (0.410 - 0.527) | 0.452 (0.396 - 0.509) | 0.438 (0.384 - 0.494) | 0.416 (0.364 - 0.470) | 0.419 (0.367 - 0.472) | 0.412 (0.361 - 0.465) | 0.416 (0.365 - 0.469) | 0.405 (0.355 - 0.457) | 0.404 (0.354 - 0.456) | 0.396 (0.347 - 0.447) |
|  |  | Step2  (After  -Triage) | class ratio (%) | | | 0.41 | 0.58 | 0.70 | 0.79 | 0.88 | 0.94 | 0.98 | 1.02 | 1.07 | 1.11 | 1.14 | 1.18 |
| Uni  modal  _C | Logistic  regression |  | AUC | 0.892 | 0.009 | 0.913 (0.903 - 0.923) | 0.906 (0.897 - 0.915) | 0.901 (0.893 - 0.909) | 0.895 (0.887 - 0.903) | 0.888 (0.880 - 0.896) | 0.886 (0.879 - 0.893) | 0.885 (0.878 - 0.892) | 0.886 (0.879 - 0.893) | 0.885 (0.878 - 0.892) | 0.887 (0.880 - 0.894) | 0.887 (0.880 - 0.894) | 0.887 (0.880 - 0.894) |
|  |  |  | APR | 0.125 | 0.003 | 0.125 (0.109 - 0.143) | 0.132 (0.118 - 0.147) | 0.126 (0.114 - 0.140) | 0.124 (0.112 - 0.137) | 0.121 (0.110 - 0.133) | 0.120 (0.109 - 0.131) | 0.120 (0.110 - 0.131) | 0.123 (0.113 - 0.134) | 0.124 (0.114 - 0.135) | 0.126 (0.116 - 0.137) | 0.126 (0.116 - 0.137) | 0.128 (0.067 - 0.232) |
| Uni  modal_B | Unimodal  Transformer |  | AUC | 0.938 | 0.011 | 0.962 (0.955 - 0.969) | 0.952 (0.946 - 0.958) | 0.947 (0.941 - 0.953) | 0.942 (0.936 - 0.948) | 0.936 (0.930 - 0.942) | 0.934 (0.928 - 0.940) | 0.932 (0.926 - 0.938) | 0.931 (0.925 - 0.937) | 0.930 (0.924 - 0.936) | 0.929 (0.923 - 0.935) | 0.928 (0.922 - 0.934) | 0.928 (0.923 - 0.933) |
|  |  |  | APR | 0.228 | 0.014 | 0.249 (0.228 - 0.272) | 0.254 (0.236 - 0.273) | 0.243 (0.227 - 0.260) | 0.236 (0.221 - 0.252) | 0.228 (0.214 - 0.243) | 0.226 (0.212 - 0.240) | 0.219 (0.206 - 0.233) | 0.220 (0.207 - 0.234) | 0.217 (0.204 - 0.230) | 0.217 (0.204 - 0.230) | 0.215 (0.203 - 0.228) | 0.215 (0.203 - 0.228) |
|  |  |  | class ratio (%) | | | 0.40 | 0.56 | 0.68 | 0.77 | 0.85 | 0.91 | 0.95 | 0.99 | 1.04 | 1.08 | 1.11 | 1.15 |
| Tri modal | Multimodal  Transformer |  | AUC | 0.942 | 0.011 | 0.967 (0.961 - 0.973) | 0.957 (0.951 - 0.963) | 0.951 (0.945 - 0.957) | 0.947 (0.941 - 0.953) | 0.940 (0.934 - 0.946) | 0.937 (0.931 - 0.943) | 0.936 (0.931 - 0.941) | 0.935 (0.930 - 0.940) | 0.933 (0.928 - 0.938) | 0.933 (0.928 - 0.938) | 0.932 (0.927 - 0.937) | 0.932 (0.927 - 0.937) |
|  |  |  | APR | 0.235 | 0.016 | 0.251 (0.230 - 0.273) | 0.266 (0.248 - 0.285) | 0.255 (0.239 - 0.272) | 0.244 (0.229 - 0.260) | 0.234 (0.220 - 0.249) | 0.230 (0.216 - 0.244) | 0.227 (0.214 - 0.241) | 0.224 (0.211 - 0.237) | 0.224 (0.211 - 0.237) | 0.220 (0.208 - 0.233) | 0.221 (0.209 - 0.234) | 0.220 (0.208 - 0.232) |

(B)

| **Model**  **architecture** | |  |  | **Multi Within Ranges Prediction Performance** | | | | | | | | | | | | | |
| --- | --- | --- | --- | --- | --- | --- | --- | --- | --- | --- | --- | --- | --- | --- | --- | --- | --- |
| **data** | **model** | **Prediction**  **time** |  | **mean** | **std** | **0-1** | **0-2** | **0-3** | **0-4** | **0-5** | **0-6** | **0-7** | **0-8** | **0-9** | **0-10** | **0-11** | **0-12** |
|  |  | Step1  (Triage) | class ratio (%) | | | 0.31 | 0.71 | 1.06 | 1.33 | 1.54 | 1.72 | 1.86 | 1.98 | 2.08 | 2.16 | 2.23 | 2.29 |
| Uni  modal_A | Logistic  regression |  | AUC | 0.952 | 0.011 | 0.971 (0.952 - 0.990) | 0.968 (0.955 - 0.981) | 0.964 (0.953 - 0.975) | 0.962 (0.952 - 0.972) | 0.954 (0.943 - 0.965) | 0.950 (0.940 - 0.960) | 0.948 (0.938 - 0.958) | 0.946 (0.937 - 0.957) | 0.945 (0.935 - 0.955) | 0.942 (0.932 - 0.952) | 0.941 (0.931 - 0.951) | 0.939 (0.929 - 0.949) |
|  |  |  | APR | 0.295 | 0.047 | 0.167 (0.115 - 0.236) | 0.239 (0.196 - 0.288) | 0.285 (0.247 - 0.326) | 0.306 (0.271 - 0.343) | 0.308 (0.276 - 0.343) | 0.312 (0.281 - 0.345) | 0.314 (0.284 - 0.345) | 0.317 (0.288 - 0.347) | 0.321 (0.293 - 0.351) | 0.321 (0.293 - 0.350) | 0.324 (0.296 - 0.353) | 0.321 (0.294 - 0.349) |
| Bi  modal_A | Multimodal  Transformer |  | AUC | 0.965 | 0.010 | 0.979 (0.963 - 0.995) | 0.979 (0.968 - 0.990) | 0.975 (0.965 - 0.985) | 0.974 (0.965 - 0.983) | 0.967 (0.958 - 0.976) | 0.963 (0.954 - 0.972) | 0.961 (0.952 - 0.970) | 0.959 (0.950 - 0.968) | 0.957 (0.948 - 0.966) | 0.955 (0.946 - 0.964) | 0.953 (0.944 - 0.962) | 0.952 (0.943 - 0.961) |
|  |  |  | APR | 0.385 | 0.038 | 0.278 (0.212 - 0.355) | 0.344 (0.295 - 0.397) | 0.386 (0.344 - 0.429) | 0.402 (0.364 - 0.441) | 0.398 (0.363 - 0.434) | 0.401 (0.368 - 0.435) | 0.399 (0.367 - 0.432) | 0.400 (0.369 - 0.432) | 0.402 (0.372 - 0.433) | 0.403 (0.373 - 0.433) | 0.403 (0.374 - 0.433) | 0.407 (0.378 - 0.437) |
|  |  | Step2  (After  -Triage) | class ratio (%) | | | 1.17 | 2.12 | 2.81 | 3.27 | 3.64 | 3.91 | 4.13 | 4.32 | 4.47 | 4.58 | 4.68 | 4.77 |
| Uni  modal  _C | Logistic  regression |  | AUC | 0.894 | 0.014 | 0.920 (0.914 - 0.926) | 0.915 (0.911 - 0.919) | 0.908 (0.904 - 0.912) | 0.901 (0.897 - 0.905) | 0.895 (0.891 - 0.899) | 0.891 (0.887 - 0.895) | 0.888 (0.884 - 0.892) | 0.886 (0.882 - 0.890) | 0.885 (0.881 - 0.889) | 0.883 (0.880 - 0.886) | 0.881 (0.878 - 0.884) | 0.880 (0.877 - 0.883) |
|  |  |  | APR | 0.273 | 0.038 | 0.166 (0.155 - 0.178) | 0.238 (0.228 - 0.248) | 0.264 (0.255 - 0.273) | 0.274 (0.266 - 0.282) | 0.282 (0.274 - 0.290) | 0.285 (0.277 - 0.293) | 0.289 (0.282 - 0.297) | 0.293 (0.286 - 0.300) | 0.295 (0.288 - 0.302) | 0.296 (0.289 - 0.303) | 0.296 (0.289 - 0.303) | 0.297 (0.290 - 0.304) |
| Uni  modal_B | Unimodal  Transformer |  | AUC | 0.945 | 0.012 | 0.966 (0.962 - 0.970) | 0.962 (0.959 - 0.965) | 0.956 (0.953 - 0.959) | 0.951 (0.948 - 0.954) | 0.947 (0.944 - 0.950) | 0.943 (0.940 - 0.946) | 0.940 (0.937 - 0.943) | 0.938 (0.935 - 0.941) | 0.937 (0.934 - 0.940) | 0.935 (0.932 - 0.938) | 0.933 (0.930 - 0.936) | 0.932 (0.929 - 0.935) |
|  |  |  | APR | 0.428 | 0.059 | 0.258 (0.245 - 0.272) | 0.381 (0.370 - 0.392) | 0.421 (0.411 - 0.431) | 0.433 (0.424 - 0.442) | 0.440 (0.431 - 0.449) | 0.447 (0.439 - 0.455) | 0.453 (0.445 - 0.461) | 0.458 (0.450 - 0.466) | 0.460 (0.452 - 0.468) | 0.461 (0.453 - 0.469) | 0.462 (0.454 - 0.470) | 0.463 (0.455 - 0.471) |
|  |  |  | class ratio (%) | | | 1.13 | 2.04 | 2.70 | 3.15 | 3.51 | 3.77 | 3.99 | 4.16 | 4.31 | 4.42 | 4.52 | 4.60 |
| Tri modal | Multimodal  Transformer |  | AUC | 0.942 | 0.012 | 0.964 (0.960 - 0.968) | 0.960 (0.957 - 0.963) | 0.954 (0.951 - 0.957) | 0.949 (0.946 - 0.952) | 0.944 (0.941 - 0.947) | 0.940 (0.937 - 0.943) | 0.937 (0.934 - 0.940) | 0.935 (0.932 - 0.938) | 0.934 (0.931 - 0.937) | 0.932 (0.929 - 0.935) | 0.930 (0.927 - 0.933) | 0.928 (0.925 - 0.931) |
|  |  |  | APR | 0.422 | 0.053 | 0.268 (0.255 - 0.281) | 0.382 (0.371 - 0.393) | 0.416 (0.407 - 0.426) | 0.428 (0.419 - 0.437) | 0.434 (0.426 - 0.442) | 0.439 (0.431 - 0.447) | 0.444 (0.436 - 0.452) | 0.449 (0.441 - 0.457) | 0.453 (0.445 - 0.461) | 0.453 (0.445 - 0.461) | 0.451 (0.444 - 0.458) | 0.450 (0.443 - 0.457) |

(C)

| **Model**  **architecture** | |  |  | **Multi Within Ranges Prediction Performance** | | | | | | | | | | | | | |
| --- | --- | --- | --- | --- | --- | --- | --- | --- | --- | --- | --- | --- | --- | --- | --- | --- | --- |
| **data** | **model** | **Prediction**  **time** |  | **mean** | **std** | **0-1** | **0-2** | **0-3** | **0-4** | **0-5** | **0-6** | **0-7** | **0-8** | **0-9** | **0-10** | **0-11** | **0-12** |
|  |  | Step1  (Triage) | class ratio (%) | | | 0.26 | 0.81 | 1.23 | 1.58 | 1.94 | 2.23 | 2.56 | 2.76 | 2.92 | 3.06 | 3.17 | 3.25 |
| Uni  modal_A | Logistic  regression |  | AUC | 0.878 | 0.016 | 0.912 (0.877 - 0.947) | 0.907 (0.887 - 0.927) | 0.888 (0.870 - 0.906) | 0.877 (0.861 - 0.893) | 0.873 (0.858 - 0.888) | 0.871 (0.857 - 0.885) | 0.869 (0.856 - 0.882) | 0.867 (0.854 - 0.880) | 0.868 (0.856 - 0.880) | 0.867 (0.855 - 0.879) | 0.867 (0.855 - 0.879) | 0.867 (0.855 - 0.879) |
|  |  |  | APR | 0.141 | 0.054 | 0.022 (0.007 - 0.070) | 0.073 (0.051 - 0.104) | 0.104 (0.082 - 0.132) | 0.117 (0.096 - 0.142) | 0.134 (0.113 - 0.158) | 0.146 (0.126 - 0.169) | 0.166 (0.146 - 0.188) | 0.172 (0.153 - 0.193) | 0.183 (0.163 - 0.204) | 0.188 (0.169 - 0.209) | 0.191 (0.172 - 0.212) | 0.194 (0.175 - 0.215) |
| Bi  modal_A | Multimodal  Transformer |  | AUC | 0.916 | 0.019 | 0.956 (0.931 - 0.981) | 0.946 (0.930 - 0.962) | 0.935 (0.921 - 0.949) | 0.918 (0.904 - 0.932) | 0.911 (0.898 - 0.924) | 0.906 (0.894 - 0.918) | 0.905 (0.894 - 0.916) | 0.903 (0.892 - 0.914) | 0.903 (0.892 - 0.914) | 0.903 (0.892 - 0.914) | 0.905 (0.895 - 0.915) | 0.905 (0.895 - 0.915) |
|  |  |  | APR | 0.258 | 0.063 | 0.079 (0.043 - 0.142) | 0.231 (0.192 - 0.276) | 0.249 (0.216 - 0.286) | 0.244 (0.215 - 0.276) | 0.251 (0.224 - 0.280) | 0.261 (0.235 - 0.288) | 0.276 (0.252 - 0.302) | 0.282 (0.258 - 0.307) | 0.292 (0.269 - 0.317) | 0.301 (0.278 - 0.325) | 0.310 (0.287 - 0.334) | 0.316 (0.293 - 0.340) |
|  |  | Step2  (After  -Triage) | class ratio (%) | | | 1.05 | 1.92 | 2.61 | 3.19 | 3.69 | 4.14 | 4.45 | 4.68 | 4.87 | 5.02 | 5.11 | 5.20 |
| Uni  modal  _C | Logistic  regression |  | AUC | 0.806 | 0.004 | 0.817 (0.809 - 0.825) | 0.812 (0.806 - 0.818) | 0.807 (0.802 - 0.812) | 0.806 (0.801 - 0.811) | 0.804 (0.799 - 0.809) | 0.805 (0.801 - 0.809) | 0.804 (0.800 - 0.808) | 0.803 (0.799 - 0.807) | 0.803 (0.799 - 0.807) | 0.803 (0.799 - 0.807) | 0.804 (0.800 - 0.808) | 0.804 (0.800 - 0.808) |
|  |  |  | APR | 0.155 | 0.046 | 0.055 (0.048 - 0.063) | 0.092 (0.085 - 0.099) | 0.118 (0.112 - 0.125) | 0.137 (0.131 - 0.143) | 0.152 (0.146 - 0.158) | 0.167 (0.161 - 0.173) | 0.177 (0.171 - 0.183) | 0.183 (0.177 - 0.189) | 0.188 (0.182 - 0.194) | 0.193 (0.187 - 0.199) | 0.196 (0.190 - 0.202) | 0.200 (0.194 - 0.206) |
| Uni  modal_B | Unimodal  Transformer |  | AUC | 0.870 | 0.010 | 0.895 (0.888 - 0.902) | 0.885 (0.880 - 0.890) | 0.874 (0.869 - 0.879) | 0.870 (0.866 - 0.874) | 0.866 (0.862 - 0.870) | 0.865 (0.861 - 0.869) | 0.865 (0.861 - 0.869) | 0.865 (0.861 - 0.869) | 0.865 (0.862 - 0.868) | 0.865 (0.862 - 0.868) | 0.865 (0.862 - 0.868) | 0.865 (0.862 - 0.868) |
|  |  |  | APR | 0.238 | 0.051 | 0.123 (0.113 - 0.134) | 0.175 (0.166 - 0.184) | 0.195 (0.187 - 0.203) | 0.219 (0.212 - 0.227) | 0.235 (0.228 - 0.242) | 0.251 (0.244 - 0.258) | 0.264 (0.257 - 0.271) | 0.269 (0.262 - 0.276) | 0.277 (0.270 - 0.284) | 0.280 (0.274 - 0.287) | 0.284 (0.278 - 0.291) | 0.287 (0.281 - 0.294) |
|  |  |  | class ratio (%) | | | 1.02 | 1.87 | 2.53 | 3.08 | 3.56 | 3.99 | 4.28 | 4.50 | 4.68 | 4.83 | 4.92 | 5.00 |
| Tri modal | Multimodal  Transformer |  | AUC | 0.875 | 0.007 | 0.893 (0.886 - 0.900) | 0.887 (0.882 - 0.892) | 0.879 (0.875 - 0.883) | 0.873 (0.869 - 0.877) | 0.871 (0.867 - 0.875) | 0.870 (0.866 - 0.874) | 0.870 (0.867 - 0.873) | 0.871 (0.868 - 0.874) | 0.872 (0.869 - 0.875) | 0.872 (0.869 - 0.875) | 0.873 (0.870 - 0.876) | 0.874 (0.871 - 0.877) |
|  |  |  | APR | 0.259 | 0.061 | 0.129 (0.119 - 0.140) | 0.186 (0.177 - 0.195) | 0.209 (0.201 - 0.217) | 0.228 (0.221 - 0.235) | 0.249 (0.242 - 0.256) | 0.269 (0.262 - 0.276) | 0.284 (0.277 - 0.291) | 0.293 (0.286 - 0.300) | 0.304 (0.298 - 0.311) | 0.313 (0.307 - 0.320) | 0.318 (0.312 - 0.324) | 0.324 (0.318 - 0.330) |
| Unimodal_A: [Gender, Age, KTAS], [vital sign_initial (SBP, HR, RR, BT, Sat), Consciousness_initial] Bimodal_A : [Gender, Age, KTAS], [vital sign_initial (SBP, DBP, HR, RR, BT, Sat), Consciousness_initial] + Present illness (text) Unimodal_B: [Gender, Age, KTAS], [vital sign + lab test, Consciousness (time-series)] Bimodal_B : [Gender, Age, KTAS], [vital sign + lab test, Consciousness (time-series)] + Present illness (text) Trimodal : [Gender, Age, KTAS], [vital sign + lab test, Consciousness (time-series)] + Present illness (text) + CXR (image) | | | | | | | | | | | | | | | | | |
|  |  |  |  |  |  |  |  |  |  |  |  |  |  |  |  |  |  |
|  |  |  |  |  |  |  |  |  |  |  |  |  |  |  |  |  |  |

(D)

**Supplementary table 2. The AUROC and AUPRC scores alongside the time resolution of input data with 95% confidence interval**

|  | **Sparse data** | **30** | **25** | **20** | **15** | **10** | **5** | **1** |
| --- | --- | --- | --- | --- | --- | --- | --- | --- |
| AUROC | 0.551 (0.489 - 0.614) | 0.805 (0.752 - 0.858) | 0.801 (0.747 - 0.854) | 0.768 (0.711 - 0.824) | 0.774 (0.718 - 0.830) | 0.760 (0.703 - 0.817) | 0.774 (0.718 - 0.830) | 0.899 (0.858 - 0.940) |
| AUPRC | 0.137 (0.083 - 0.218) | 0.310 (0.228 - 0.406) | 0.354 (0.268 - 0.451) | 0.332 (0.248 - 0.429) | 0.371 (0.283 - 0.468) | 0.342 (0.257 - 0.439) | 0.410 (0.319 - 0.507) | 0.541 (0.444 - 0.634) |

AUROC; Area Under the Receiver Operating Characteristic Curve, AUPRC: Area Under the Precision-Recall Curve

**Supplementary table 3. Step 2 Logistic regression model**

|  | | |  |  | Validation | | | | Test | | | | |
| --- | --- | --- | --- | --- | --- | --- | --- | --- | --- | --- | --- | --- | --- |
| Modality | Text  Types | Target | lr_initial | seed | apr | auc | avg_apr | avg_auc | apr | auc | apr+auc | avg_apr | avg_auc |
| Uni  modal |  | In  hospital  cardiac  arrest | 1.00E-04 | 0 | 0.047 | 0.911 | 0.054 | 0.923 | 0.036 | 0.875 | 0.911 | 0.043 | 0.886 |
|  |  |  |  | 1004 | 0.062 | 0.937 |  |  | 0.041 | 0.904 | 0.945 |  |  |
|  |  |  |  | 2022 | 0.054 | 0.921 |  |  | 0.052 | 0.880 | 0.931 |  |  |
|  |  | Advanced  airway | 3.00E-05 | 0 | 0.298 | 0.950 | 0.299 | 0.952 | 0.222 | 0.937 | 1.160 | 0.228 | 0.938 |
|  |  |  |  | 1004 | 0.294 | 0.953 |  |  | 0.234 | 0.939 | 1.173 |  |  |
|  |  |  |  | 2022 | 0.306 | 0.953 |  |  | 0.228 | 0.938 | 1.166 |  |  |
|  |  | Circulatory shock | 3.00E-05 | 0 | 0.440 | 0.946 | 0.435 | 0.946 | **0.428** | **0.945** | **1.373** | 0.419 | 0.945 |
|  |  |  |  | 1004 | 0.430 | 0.945 |  |  | 0.416 | 0.945 | 1.361 |  |  |
|  |  |  |  | 2022 | 0.434 | 0.947 |  |  | 0.413 | 0.944 | 1.357 |  |  |
|  |  | ICU admission | 1.00E-05 | 0 | 0.246 | 0.873 | 0.242 | 0.872 | 0.238 | 0.870 | 1.109 | 0.237 | 0.870 |
|  |  |  |  | 1004 | 0.241 | 0.871 |  |  | 0.239 | 0.870 | 1.109 |  |  |
|  |  |  |  | 2022 | 0.240 | 0.872 |  |  | 0.234 | 0.871 | 1.105 |  |  |
| Bi  modal | PI | In  hospital  cardiac  arrest | 1.00E-04 | 0 | 0.041 | 0.925 | 0.051 | 0.931 | 0.037 | 0.885 | 0.922 | 0.043 | 0.891 |
|  |  |  |  | 1004 | 0.047 | 0.935 |  |  | 0.044 | 0.891 | 0.934 |  |  |
|  |  |  |  | 2022 | 0.065 | 0.931 |  |  | 0.048 | 0.898 | 0.946 |  |  |
|  | PI  without  encoder | Advanced  airway | 1.00E-05 | 0 | 0.280 | 0.944 | 0.279 | 0.942 | 0.196 | 0.934 | 1.130 | 0.194 | 0.931 |
|  |  |  |  | 1004 | 0.262 | 0.943 |  |  | 0.195 | 0.930 | 1.126 |  |  |
|  |  |  |  | 2022 | 0.295 | 0.940 |  |  | 0.190 | 0.929 | 1.119 |  |  |
|  | PI | Circulatory shock | 1.00E-05 | 0 | 0.405 | 0.937 | 0.414 | 0.939 | 0.411 | 0.941 | 1.352 | 0.410 | 0.941 |
|  |  |  |  | 1004 | 0.423 | 0.942 |  |  | 0.424 | 0.942 | 1.366 |  |  |
|  |  |  |  | 2022 | 0.412 | 0.940 |  |  | 0.395 | 0.939 | 1.334 |  |  |
|  | PI+EKG | ICU admission | 1.00E-05 | 0 | 0.271 | 0.880 | 0.279 | 0.882 | 0.248 | 0.874 | 1.122 | 0.252 | 0.877 |
|  |  |  |  | 1004 | 0.275 | 0.883 |  |  | 0.245 | 0.876 | 1.120 |  |  |
|  |  |  |  | 2022 | 0.291 | 0.885 |  |  | **0.263** | **0.881** | **1.144** |  |  |
| Tri  modal | PI | In  hospital  cardiac  arrest | 1.00E-04 | 0 | 0.040 | 0.917 | 0.052 | 0.917 | 0.054 | 0.889 | 0.943 | 0.053 | 0.884 |
|  |  |  |  | 1004 | 0.069 | 0.935 |  |  | **0.053** | **0.891** | **0.945** |  |  |
|  |  |  |  | 2022 | 0.046 | 0.901 |  |  | 0.051 | 0.871 | 0.922 |  |  |
|  | PI  without  encoder | Advanced  airway | 3.00E-05 | 0 | 0.289 | 0.950 | 0.294 | 0.953 | 0.238 | 0.939 | 1.177 | 0.238 | 0.941 |
|  |  |  |  | 1004 | 0.300 | 0.954 |  |  | **0.235** | **0.942** | **1.176** |  |  |
|  |  |  |  | 2022 | 0.295 | 0.955 |  |  | 0.241 | 0.943 | 1.184 |  |  |
|  | PI | Circulatory shock | 1.00E-05 | 0 | 0.430 | 0.945 | 0.430 | 0.944 | 0.419 | 0.942 | 1.362 | 0.424 | 0.943 |
|  |  |  |  | 1004 | 0.431 | 0.944 |  |  | 0.422 | 0.942 | 1.364 |  |  |
|  |  |  |  | 2022 | 0.430 | 0.944 |  |  | 0.430 | 0.944 | 1.373 |  |  |
|  | PI+EKG | ICU admission | 1.00E-05 | 0 | 0.285 | 0.880 | 0.288 | 0.882 | 0.270 | 0.874 | 1.144 | 0.267 | 0.876 |
|  |  |  |  | 1004 | 0.288 | 0.882 |  |  | 0.273 | 0.878 | 1.151 |  |  |
|  |  |  |  | 2022 | 0.290 | 0.884 |  |  | 0.259 | 0.875 | 1.134 |  |  |

PI; present illness, AUC; Area Under the Receiver Operating Characteristic Curve, APR: Area Under the Precision-Recall Curve, EKG; electrocardiogram
